# A vulnerable age group: the impact of cancer on the psychosocial well-being of young adult childhood cancer survivors

**DOI:** 10.1007/s00520-021-06009-y

**Published:** 2021-02-01

**Authors:** L. M. E. van Erp, H. Maurice-Stam, L. C. M. Kremer, W. J. E. Tissing, H. J. H. van der Pal, A. C. H. de Vries, M. M. van den Heuvel-Eibrink, B. A. B. Versluys, M. van der Heiden-van der Loo, G. A. Huizinga, M. A. Grootenhuis

**Affiliations:** 1 Princess Máxima Center for Pediatric Oncology, Heidelberglaan 25, 3584CS Utrecht, The Netherlands; 2 Emma Children’s Hospital, Amsterdam UMC, University of Amsterdam, Amsterdam, The Netherlands; 3 Beatrix Children’s Hospital/University of Groningen/University Medical Center Groningen, Groningen, The Netherlands; 4 Sophia Children’s Hospital/Erasmus Medical Center, Rotterdam, The Netherlands; 5 Wilhelmina Children’s Hospital/University Medical Center Utrecht, Utrecht, The Netherlands; 6 Dutch Childhood Oncology Group, Utrecht, The Netherlands

**Keywords:** Childhood cancer, Young adults, Quality of life, Anxiety, Depression, Fatigue

## Abstract

**Purpose:**

This study aimed to increase our understanding of the psychosocial well-being of young adult childhood cancer survivors (YACCS) as well as the positive and negative impacts of cancer.

**Methods:**

YACCS (aged 18–30, diagnosed ≤ 18, time since diagnosis ≥ 5 years) cross-sectionally filled out the “Pediatric Quality of Life Inventory Young Adults” (PedsQL-YA), “Hospital Anxiety and Depression Scale” (HADS), and “Checklist Individual Strengths” (CIS-20R) to measure fatigue and survivor-specific “Impact of Cancer - Childhood Survivors” (IOC-CS), which measures the long-term impact of childhood cancer in several domains. Descriptive statistics (IOC-CS), logistic regression (HADS, CIS-20R), and ANOVA (PedsQL-YA, HADS, CIS-20R) were performed. Associations between positive and negative impacts of childhood cancer and psychosocial outcomes were examined with linear regression analyses.

**Results:**

YACCS (*N* = 151, 61.6% female, mean age 24.1 ± 3.6, mean time since diagnosis 13.6 ± 3.8) reported lower HRQOL (− .4 ≤ *d* ≤ − .5, *p* ≤ .001) and more anxiety (*d* = .4, *p* ≤ .001), depression (*d* = .4, *p* ≤ .01), and fatigue (.3 ≤ *d* ≤ .5, *p* ≤ .001) than young adults from the general Dutch population. They were at an increased risk of experiencing (sub)clinical anxiety (OR = 1.8, *p* = .017). YACCS reported more impact on scales representing a positive rather than negative impact of CC. Various domains of impact of childhood cancer were related to psychosocial outcomes, especially “Life Challenges” (HRQOL *β* = − .18, anxiety *β* = .36, depression *β* = .29) and “Body & Health” (HRQOL *β* = .27, anxiety *β* = − .25, depression *β* = − .26, fatigue *β* = − .47).

**Conclusion:**

YACCS are vulnerable to psychosocial difficulties, but they also experience positive long-term impacts of childhood cancer. Positive and negative impacts of childhood cancer were associated with psychosocial outcomes in YACCS. Screening of psychosocial outcomes and offering targeted interventions are necessary to optimize psychosocial long-term follow-up care for YACCS.

## Background

With the survival rate of childhood cancer rising, researchers and clinicians have an increased interest in the late effects of treatment. Long-term physical morbidity is high for childhood cancer survivors (CCS) [1, 2], as well as difficulty with psychological well-being [3–5].

Looking at the well-being of a patient population within the framework of a biopsychosocial model can be beneficial when studying the role of physical, psychological, and social factors [6]. In the biopsychosocial model, behavioral and social circumstances can influence the emergence, course, and experience of a disease, while the disease in itself influences psychological well-being and social relationships [6]. In accordance with the biopsychosocial model, knowledge of psychosocial late effects is crucial for improving life beyond childhood cancer. Attention to psychosocial late effects may be especially important for young adult childhood cancer survivors (YACCS) as young adulthood is a crucial life phase with many developmental challenges to overcome, e.g., relationships, sexuality, cognition, education, employment, and developing autonomy. A life-threatening disease such as cancer can disrupt this crucial development. This seems to be confirmed by research, as overall, YACCS reach fewer developmental milestones than young adults without a history of childhood cancer, which negatively affects their quality of life [7, 8]. Both age-specific challenges and their potential disruption due to cancer can be seen within biological, psychological, and social domains, and can often present in multiple domains.

Contradicting findings have been reported on health-related quality of life (HRQOL) and well-being of CCS [3, 9–17]. Most (young) adult CCS did not report psychopathology, but survivors of central nervous system (CNS) tumors, those treated with cranial irradiation, and those with chronic health conditions had worse outcomes (distress, anxiety, depression, somatization, HRQOL, mental health dysfunction, fatigue, PTSD, unemployment, educational attainment) compared to reference groups [3, 4, 12, 13, 17–23]. While some studies indicate that fatigue is a problem among (YA)CCS [18, 20], other studies show that fatigue levels among CCS do not differ from reference groups or that clinical significance is questionable [24, 25]. A recent review concluded that the prevalence of severe fatigue among CCS remains unclear, due in part to the heterogeneity of studies regarding inclusion criteria and samples as well as the questionnaires used to assess fatigue [26]. As fatigue has previously been linked to poor (HR)QOL [5, 18, 27], it is crucial to investigate the incidence of fatigue in the Dutch population of YACCS and explore underlying mechanisms.

While almost all CCS studies include YACCS, most research among CCS does not differentiate between children, young adults, and older adults. Researching YACCS separately from older and younger CCS is crucial in order to understand the specific vulnerabilities and needs of young adults, which is necessary to provide CCS with targeted interventions that may help them bridge the gap between themselves and healthy peers early in their lives.

Besides the distinction of YACCS from both younger and older CCS, it is important to study YACCS separately from patients with and survivors of adolescent and young adult (AYA(-S)) cancer. While YACCS and AYA(-S) may be the same age, YACCS distinguish themselves regarding their diagnosis and treatment, a longer time since diagnosis, the presence of late effects of treatment, and a possible lack of knowledge about both their medical history and risk of late effects because of missed information during childhood. AYA cancer patients and YACCS are sometimes studied as one group, while results for the one group are not generalizable to the other [28, 29]. Survivors of cancer in the AYA age report challenges (i.e., financial independence and protecting parents, cognitive decline in case of a brain tumor). These challenges differ from those reported by YACCS: identity formation, social isolation, health care transitions, and for those diagnosed with a brain tumor: cognitive deficits, limited career options, poor social skills. However, the two groups also express common challenges, such as physical appearance, fertility, late effects, social relationships, and changing priorities [30].

In order to increase our understanding of the experiences of and challenges for YACCS (aged 18–30, diagnosis at age < 18), it is of great importance to look further than generic psychosocial constructs. Taking survivor-specific psychosocial factors into account can yield a broader perspective on the functioning of YACCS, which may help us tailor interventions to their needs. To gain broad insight into this functioning, the present study focused on generic psychosocial well-being, psychopathology, and survivor-specific constructs. First, to align with the previous literature, this study aimed to describe generic HRQOL, depression, anxiety, and fatigue in Dutch YACCS in comparison with reference groups. Secondly, the study aimed to describe the perceived impact of CC, both positive and negative. By examining this survivor-specific construct, the authors aimed to deepen our insight into the experiences of YACCS. Finally, the study aimed to investigate the role of the survivor-specific construct of perceived impact in explaining generic psychosocial outcomes and psychopathology (HRQOL, depression, anxiety, and fatigue) in Dutch YACCS controlled for sociodemographic and medical characteristics.

## Methods

A total of 400 YACCS were selected by a data manager of the Dutch LATER registry from 946 YACCS who met the eligibility criteria for the study (aged 18–30, diagnosed at age < 18, ≥ 5 years since diagnosis, treated at one of the four participating Dutch pediatric oncology centers, and no participation in the Dutch LATER study in the past 4 months) in the pseudonymized Dutch LATER registry. The selection was stratified in order to have an equal representation of men and women between the ages of 18 to 24 and 25 to 30, as well as various groups based on age at diagnosis.

A total of 22 YACCS were excluded from the invitation for being recently deceased, having no known address, or living abroad. In 2018, the 378 remaining eligible YACCS were invited to fill out questionnaires on paper or online. Participants provided written informed consent and the Medical Ethical Committee of the University Hospital Utrecht reviewed this study (case number 18/256).

### Measures

#### Medical characteristics

Diagnosis and treatment data on the initial childhood cancer and recurrences, as well as aggregated data for non-participants, was collected from the Dutch LATER registry.

#### Sociodemographic characteristics

Date of birth, gender, marital status, number of children, employment, and educational level (attained and current) were acquired.

#### HRQOL

The Pediatric Quality of Life Inventory Young Adults (PedsQL-YA) measures HRQOL in four scales (Physical Functioning: 8 items, Cronbach’s *α* = .86; Emotional Functioning: 5 items, Cronbach’s *α* = .84; Social Functioning: 5 items, Cronbach’s *α* = .85; and Work/School Functioning: 5 items, Cronbach’s *α* = .80), a total scale (all 23 items, Cronbach’s *α* = .92), and a Psychosocial Summary Scale (PSY) combining emotional, social, and work/school functioning (15 items, Cronbach’s *α* = .90). Higher scores (range 0–100) indicate better HRQOL. The PedsQL-YA has good psychometric properties and a reference group of Dutch young adults is available [31].

#### Anxiety and depression

The Hospital Anxiety and Depression Scale (HADS) measures anxiety and depression in separate scales and a total scale [32]. Participants are asked to respond to 14 statements, seven about anxiety (Cronbach’s *α* = .88) and seven about depression (Cronbach’s *α* = .85) by selecting one of four reactions specific to that statement. Higher scores on the HADS signal higher levels of anxiety and depression. Scale scores ≥ 8 for anxiety and depression are considered (sub)clinical. The HADS has good psychometric properties [33] and a reference group of Dutch young adults is available [34].

#### Fatigue

The Checklist Individual Strength (CIS-20R) is a valid measure of fatigue, consisting of four scales: Fatigue Severity (8 items, Cronbach’s *α* = .79), Concentration (5 items, Cronbach’s *α* = .91), Motivation (4 items, Cronbach’s *α* = .82), and Activity (3 items, Cronbach’s *α* = .90). In this study, the total score was not used, as its meaning is unclear [35]. Higher scores reflect more fatigue and fatigue-related impairment. The CIS-20R has good psychometric properties and a reference group of Dutch young adults is available [35].

#### Impact of cancer

The IOC-CS is a survivor-specific questionnaire that measures perceived negative and positive impacts of CC [36]. It includes five positive impact scales (Socializing: 3 items, Cronbach’s *α* = .59; Talking with parents: 4 items, Cronbach’s *α* = .92; Body & Health: 8 items, Cronbach’s *α* = .80; Health Literacy: 5 items, Cronbach’s *α* = .71; Personal Growth: 5 items, Cronbach’s *α* = .71) and six negative impact scales (Thinking & Memory problems: 5 items, Cronbach’s *α* = .76; Sibling Concerns: 2 items, Cronbach’s *α* = .69; Life Challenges: 12 items, Cronbach’s *α* = .86; Relationship Concerns: 7 items, Cronbach’s *α* = .65 for partnered YACCS and .63 for non-partnered YACCS; Financial Problems: 3 items, Cronbach’s *α* = .77). Higher scores indicate more positive or negative impact. The IOC-CS has been translated and back-translated into Dutch by Grootenhuis and Maurice-Stam in cooperation with the author of the original IOC-CS. The original version has good psychometric properties [36].

### Statistical analyses

To compare characteristics of participants and non-participants, one-sample *t* tests and binominal tests were used.

Differences between YACCS and the reference group were tested, controlled for age and sex, using logistic regression with odds ratio (HADS, CIS-20R) and ANOVA (PedsQL-YA, HADS, CIS-20R) with Cohen’s *d* (.2 small, .5 medium, .8 large effect size) [37]. The IOC-CS scales were analyzed descriptively, using item scores and mean item scale scores.

Associations between positive and negative impacts of cancer (IOC-CS) and psychosocial outcomes were examined with multiple linear regression analyses. Separate models were estimated for PedsQL-YA total HRQOL, HADS anxiety, HADS depression, and CIS-20R Fatigue Severity, with positive and negative impacts of cancer (the IOC-CS mean item scale scores) as independent variables, while controlling for sociodemographic (sex and education) and medical (age at diagnosis, time since diagnosis, tumor type, recurrences, treatment) characteristics. All independent variables were entered in one step in all models. A significance level of .05 was used for all analyses based on two-sided tests. To adjust for multiple comparisons, a Bonferroni correction was applied per questionnaire for the PedsQL-YA (.05/6 = .008), HADS (.05/3 = .017), and CIS-20R (.05/4 = .013).

## Results

### Sample characteristics

A total of 151 YACCS (61.6% female, mean age 24.1 ± 3.6, mean time since diagnosis 13.6 ± 3.8) completed the questionnaire (response rate = 40%). Participants were more often female and less likely to have received a bone marrow transplantation (BMT) than non-participants (Table 1).

**Table 1:** Characteristics of participants and non-participants

	Participants (*N* ≈ 151)		Non-participants (*N* = 223)		
	Mean ± SD (range)	% (*N*)	Mean ± SD (range)	% (*N*)	*p* value
Socio-demographic					
Age (years)	24.1 ± 3.6 (18–30)		24.0 ± 3.4 (18–30)		.659
Sex (female)		61.6 (93)		40.8 (90)	.000**
Marital/relationship status					
Yes		51.0 (75)			
No		49.0 (72)			
Employment status					
Paid occupation		70.9 (105)			
No paid occupation		29.1 (43)			
Educational attainment					
Low		19.3 (28)			
Middle		48.3 (70)			
High		32.4 (47)			
Current education					
Low		3.1 (2)			
Middle		27.7 (18)			
High		69.2 (45)			
Medical characteristics					
Age at diagnosis	10.5 ± 4.5 (.4–17)		10.6 ± 4.5 (0–18)		.756
Time since diagnosis	13.6 ± 3.8 (6–27)		13.5 ± 3.7 (6–28)		.652
Diagnosis					
Hematologic cancers		66.9 (101)		61.7 (142)	.119
CNS tumors		8.6 (13)		9.9 (22)	.358
Solid tumors		24.5 (37)		28.3 (63)	.173
Recurrence		13.9 (21)			
Treatment^a,b^					
Surgery (S)		61.6 (93)		63.7 (142)	.323
Chemotherapy (CT)		95.4 (144)		95.5 (213)	.522
Radiotherapy (RT)		37.1 (56)		35.0 (78)	.323
SCT/BMT		7.3 (11)		13.5 (30)	.012*
Treatment combinations^b^					
CT only		32.5 (49)			
CT + RT		6.0 (9)			
RT + S		4.6 (7)			
CT + S		30.5 (46)			
CT + S + RT		26.5 (40)			

*p < 0.05; **p < 0.01 (two-sided)

^a^More than one category possible

^b^Treatments for primary tumor and (if applicable) recurrence(s)

### Psychosocial well-being of YACCS compared to the reference group

YACCS reported lower HRQOL than the reference group on all PedsQL-YA scales (− .4 ≤ *d* ≤ − .5) as well as higher levels of anxiety (*d* = .4, *p* ≤ .001) and depression (*d* = .4, *p* = .019). YACCS were more likely to experience (sub)clinical anxiety than the reference group (29.8% vs. 18.8%, OR = 1.8). On the CIS-20R, YACCS reported increased Fatigue Severity (*d* = .5) and worse Concentration (*d* = .3) and were more likely to experience severe fatigue than the reference group (36.2% vs. 20.8%, OR = 2.4, Table 2).

**Table 2:** Psychosocial well-being of YACCS (*N* ≈ 151) versus reference groups

	YACCS				Reference group^a^				
	*M*	SD	95% CI	% (*N*)	*M*	SD	% (*N*)	Cohen’s *d*	OR
PedsQL-YA (total score)	77.7*	16.3	[75.0; 80.3]		83.9	13.1		− .5	
Physical	80.2*	19.7	[77.0; 83.3]		87.1	16.0		− .4	
Emotional	70.2*	21.2	[66.8; 73.6]		77.2	18.0		− .4	
Social	82.1*	20.0	[78.9; 85.3]		87.2	14.5		− .4	
Work/school	76.8*	19.1	[73.7; 79.8]		82.3	15.7		− .4	
Psychosocial	76.3*	16.9	[73.6; 79.0]		82.2	13.7		− .4	
HADS (total score)	9.4*	6.9	[8.3; 10.5]		7.0	5.7		.4	
Anxiety	5.9*	4.3	[5.3; 6.6]		4.4	3.5		.4	
Depression	3.5*	3.5	[2.9; 4.1]		2.6	2.8		.3	
(Sub)clinical anxiety (≥ 8)			[.2; .4]	30.2 (45)			18.8 (42)		1.8*
(Sub)clinical depression (≥ 8)			[.1; .2]	12.8 (19)			7.6 (17)		1.7
CIS-20R									
Fatigue Severity	30.6*	9.9	[29.0; 32.2]		25.2	10.4		.5	
Concentration	15.8*	7.8	[14.6; 17.1]		13.8	6.3		.3	
Motivation	10.6	5.1	[9.8; 11.5]		11.1	4.7		− .1	
Activity	9.9	5.1	[9.1; 10.7]		9.7	4.6		.1	
Severe fatigue (≥ 35)			[.3; .4]	36.2 (54)			20.8 (55)		2.4*

*M* mean, *SD* standard deviation, *OR* odds ratio

PedsQL-YA: *p < .008 (two-sided)

HADS: *p < .017 (two-sided)

CIS-20R: *p < .013 (two-sided)

^a^PedsQL-YA *N* = 649; HADS *N* = 224; CIS-20-R *N* = 264

### Positive and negative impacts of childhood cancer

On scale level, most positive impact was reported on Socializing and least on Personal Growth. On item level, least positive impact was reported on “I have a special bond with others with cancer,” “I have all the information I need,” and “I know where to find information about cancer” (Table 3).

**Table 3:** Perceived impact of cancer according to the IOC-CS

	*N*	*M*^a^	SD
Positive impact scales			
Socializing	149	4.0	.91
Do not feel left out of friends’ lives	149	4.3	1.1
Do not avoid social activities	147	4.2	1.1
Make friends easily	149	3.4	1.1
Talking with Parents	150	3.5	1.2
Mom comfortable talking about cancer	148	3.5	1.3
Can talk with dad about cancer	147	3.5	1.3
Can talk with mom about cancer	148	3.5	1.4
Dad comfortable talking about cancer	146	3.4	1.3
Body & Health	151	3.5	.7
Eat healthy diet	151	3.9	.7
Lead healthy life	151	3.9	.8
Self-confident	151	3.6	.9
Feel in control	151	3.5	1.1
Healthy as those w/o cancer	151	3.4	1.3
Believe I’m attractive	151	3.2	1.0
Like my body	151	3.2	1.0
Exercise	151	3.1	1.2
Health Literacy	151	3.3	.8
Easy to talk to doctor about cancer	151	4.1	1.0
Know who to see for med problems	151	4.0	.8
Feel doctor knows cancer effects	150	3.1	1.3
Know where to find cancer info	151	2.8	1.3
Have all cancer info I need	150	2.6	1.1
Personal Growth	150	2.8	.9
More mature than those without cancer	151	3.2	1.4
Cancer part of self	150	3.1	1.3
Learned about self	150	3.1	1.3
Good things came from cancer	150	2.9	1.3
Special bond with others with cancer	145	1.9	1.1
Negative impact scales			
Thinking/Memory	150	2.5	.9
Hard to make decisions	150	3.1	1.2
Hard time thinking	150	2.6	1.4
Trouble w/long-term memory	150	2.5	1.4
Hard to learn	150	2.3	.9
Trouble w/short-term memory	150	2.2	1.3
Sibling Concerns	141	2.3	1.1
Worry how cancer affected siblings	142	2.8	1.4
Sibling had problems related to my cancer	141	1.8	1.2
Life Challenges	151	2.1	.8
Missed out on life	149	2.8	1.5
Wonder why I got cancer	151	2.5	1.4
Worry about health	150	2.4	1.3
Wonder why I survived	151	2.4	1.5
Want to forget cancer	150	2.2	1.4
Afraid to die	150	2.1	1.4
Unsure about future	150	2.1	1.3
Worry I will die at young age	150	2.0	1.3
Cancer controls my life	148	2.0	1.2
Angry about cancer	151	1.9	1.2
Time is running out	149	1.5	1.0
Something I did caused cancer	151	1.5	1.1
Relationship Concerns total	150	1.8	.8
Partnered	77	1.7	.7
Hard to talk to partner about health problem	77	2.1	.9
Worry partner will leave if cancer returns	77	1.7	1.2
Worry about having sex with partner	77	1.4	.7
Non-partnered	73	1.9	.8
Worry about telling potential partner about fertility	73	2.2	1.3
Worry about having no relationship	73	2.2	1.2
Worry about having sex	73	1.8	1.2
Worry about telling potential partner about cancer	73	1.6	.9
Financial Problems	147	1.3	.5
Trouble getting assistance/services	147	1.5	1.0
Parents financial problems from cancer	147	1.2	.6
Financial problems from cancer	147	1.1	.6

^a^Mean item scores: 1 = “none at all”; 2 = “a little bit”; 3 = “somewhat”; 4 = “quite a bit”; 5 = “very much”

On scale level, most negative impact was reported on Thinking/Memory and least on Financial Problems. On item level, highest negative impact was reported on “It’s hard to make decisions,” “I worry about how my cancer affects my sibling,” and “I feel like I missed out on life” (Table 3).

### Associations of impact of childhood cancer with psychosocial outcomes

Two positive and two negative survivor-specific impact scales were associated with more than one psychosocial outcome (Table 4). More positive perception of Socializing was associated with better HRQOL (*β* = .24) and less depression (*β* = − .24). More positive perception of one’s Body & Health was related to better HRQOL (*β* = .27) and less anxiety (*β* = − .25), depression (*β* = − .26), and fatigue (*β* = −.47).

**Table 4:** Linear regression models for HRQOL (PedsQL-YA total score), anxiety, depression (HADS), and fatigue (CIS-20R), with positive and negative impacts of cancer (IOC-CS) as independent variables and controlling for sociodemographic and medical characteristics; *N* = 133

	HRQOL			Anxiety			Depression			Fatigue Severity		
	*β*	*B*	95% CI	*β*	*B*	95% CI	*β*	*B*	95% CI	*β*	*B*	95% CI
Sex (ref = male)	ns			.17*	1.40	[.19; 2.62]	− .16*	− 1.12	[− 2.05; − .19]	ns		
Education (ref = low)												
Middle	ns			ns			ns			ns		
High	ns			ns			ns			ns		
Age at diagnosis	ns			ns			ns			ns		
Time since diagnosis	ns			ns			ns			ns		
Diagnosis (ref = hematological)												
CNS tumor	ns			− .24*	− 3.86	[− 7.05; − .67]	ns			ns		
Solid tumor	ns			ns			ns			ns		
Recurrence	ns			ns			ns			ns		
Treatment (ref = chemotherapy only)												
Chemotherapy and radiotherapy	ns			ns			ns			ns		
Chemotherapy and surgery	ns			ns			ns			ns		
Radiotherapy and surgery	ns			ns			ns			ns		
Surgery, chemo-, and radiotherapy	ns			ns			ns			ns		
BMT (ref = no)	ns			ns			ns			ns		
Positive impact of cancer												
Socializing	.24**	4.22	[1.55; 6.89]	ns			− .24**	− .94	[− 1.60; − .29]	ns		
Talking with parents	ns			ns			ns			ns		
Body & Health	.27**	6.73	[2.65; 10.81]	− .25*	− 1.61	[− 2.91; − .30]	− .26**	− 1.40	[− 2.40; − .40]	− .47***	− 7.37	[− 10.68; − 4.06]
Health Literacy	ns			ns			.20*	.91	[.14; .69]	ns		
Personal Growth	ns			ns			ns			ns		
Negative impact of cancer	ns			ns			ns			ns		
Thinking/Memory	ns			ns			ns			ns		
Sibling Concerns	.14*	1.92	[.01; 3.82]	ns			ns			ns		
Life Challenges	− .18*	− 3.45	[− 6.79; − .11]	.36**	1.86	[.79; 2.93]	.29**	1.24	[.43; 2.06]	ns		
Relationship Concerns	− .16*	− 3.26	[− 6.43; − .08]	ns			.19*	.85	[.07; 1.63]	ns		
Financial Concerns	ns			− .26**	− 2.19	[− 3.58; − .80]	ns			ns		
*R*^2^	.662***			.513***			.572***			.458***		

*ns* non-significant, but still included in the model

*p < .05; **p < .01; ***p < .001 (two-sided)

Regarding the negative impact scales, experiencing more Life Challenges was associated with lower HRQOL (*β* = − .18), more anxiety (*β* = .36), and more depression (*β* = .29). More Relationship Concerns were associated with lower HRQOL (*β* = − .16) and more depression (*β* = .19).

## Discussion

This is one of the first studies to investigate survivor-specific psychosocial well-being in a large sample of YACCS specifically, and the first to do so in the Netherlands. This study shows that YACCS appear to be vulnerable to psychosocial difficulties. They reported worse HRQOL and more anxiety, depression, and fatigue than the reference group. Effect sizes ranged from small (depression) to large (fatigue severity). This is in accordance with findings of some earlier studies in CCS cohorts, which include YACCS but do not focus specifically on them [3, 5, 13, 15, 38–40]. Psychosocial well-being similar to that of the general population has also been reported [9–12, 16, 19, 41, 42].

Our study illuminates the experiences of YACCS regarding impact of childhood cancer. The IOC-CS scale scores in this study found more impact on concepts representing positive impact (Socializing, Talking with parents, Body & Health) than on concepts representing negative impact (Thinking & Memory problems, Life Challenges). This finding is in line with a study among CCS in the USA [36]. Survivors may have a tendency to minimize the effect of the negative aspects of their cancer experience on their current lives while maximizing the positive aspects [43, 44].

### Clinical implications

Based on our findings that YACCS report worse HRQOL and more anxiety, depression, and fatigue, the authors recommend routine psychosocial screening during long-term follow-up (LTFU). LTFU clinics need to have mechanisms, e.g., in-house psychologists or adequate referral options, in place to follow-up when screening results call for psychosocial support for a YACCS. These recommendations are in line with the existing standards of care [45]. Fatigue warrants special attention as a known late effect of treatment. In a large cohort, fatigue, as well as poor sleep and vitality, was shown to be associated with survivors’ neurocognitive functioning independent of other well-known risk factors (e.g., cranial radiation and female gender) [46], making it an important topic to be addressed by physicians, nurses, and psychosocial care providers during LTFU.

YACCS’ scores on some specific items of the IOC-CS yield important insights for psychosocial care. Item scores on Health Literacy of the IOC-CS showed that YACCS perceived a lack of information about the long-term effects of childhood cancer as well as a lack of the skills required to obtain such information. Information provision and supporting YACCS’ health literacy skills are important tasks for health care providers during LTFU. YACCS have previously reported problems with autonomy development [7] and gaining independence from their parents [47, 48], as well as with lacking information [49]. This disruption in crucial developmental areas during young adulthood could have consequences for their psychosocial well-being as well as their self-management in adulthood. YACCS may therefore benefit from a focus on patient empowerment during their LTFU.

Positive and negative impacts of childhood cancer were more strongly associated with psychosocial well-being than sociodemographic and medical characteristics (see Table 4), which Zebrack [44] also found. These findings align with earlier studies that showed the role of self-reported functional limitations and health beliefs in relation to HRQOL of CCS [42, 50] as well as a strong association between fatigue and emotional distress and functional limitations in survivors of childhood Hodgkin’s lymphoma [51]. YACCS’ perception of their Body & Health, Life Challenges, Socializing, and Relationships need special attention during LTFU based on their associations with psychosocial outcomes found in this study. While Thinking and Memory problems had the highest perceived negative impact, they were not associated with the psychosocial outcomes in our sample of YACCS. This finding is worth further exploration, because previous literature suggests that neuropsychological late effects of childhood cancer are common and can be severe [52, 53]. Furthermore, previous results showed that neuropsychological late effects can potentially influence psychosocial outcomes such as HRQOL [54–56] and fatigue [46].

Regarding the high percentages of explained variance in our models, it is arguable that perceived impact of childhood cancer and generic psychosocial outcomes are overlapping constructs. Furthermore, it is plausible to assume that the associations between impact of childhood cancer and psychosocial well-being are bidirectional. Accordingly, interventions directed at the perceived impact of cancer, e.g., cognitive behavioral therapy (CBT), could also improve psychosocial well-being, and vice versa. The value of understanding perceived impact of childhood cancer is that it may help us tailor interventions specifically to YACCS by focusing on maladaptive cognitions related to the impact of childhood cancer in young adulthood. In line with this understanding, the psychosocial department at the Princess Máxima Center has recently added an e-health module for YACCS to our CBT-based program “Op Koers” [57] and conducted a pilot. The initial results were promising. The authors’ next research efforts will focus on evaluating the intervention in order to provide this vulnerable group with an evidence-based psychosocial program.

### Study limitations

This study has some limitations that need to be taken into account. First, there was a 40% response rate, though non-participants hardly differed from participants. Compared to other studies from the Dutch LATER cohort, survivors of CNS tumors seem to be underrepresented in our study [58]. This may complicate the generalization of our study’s findings to all Dutch YACCS. Our within-group models are probably unaffected by the response rate. Second, because of the cross-sectional nature of this study, it was impossible to distinguish between cause and effect within the relationships found in our sample. Third, educational attainment was included as a predictor in our regression models because this variable was most indicative of socioeconomic status (SES) out of the data available. However, educational attainment has previously been found to be an outcome of childhood cancer history in the literature [23, 47]. Fourth, the presence of chronic health problems due to the disease were not taken into account in the regression models because we did not have access to data on disease burden.

Lastly, a limited number of independent variables were entered into our linear regression model. The aim of this study was to investigate the role of the positive and negative impact of cancer as opposed to creating the most fitting model to explain psychosocial outcomes in YACCS.

## Conclusion

YACCS are a vulnerable group. That said, they reported more positive than negative impacts of CC. The perceived impact of CC, positive as well as negative, was more strongly associated with psychosocial well-being than sociodemographic and medical characteristics. Addressing perceived impact of childhood cancer may be the gateway for targeting psychosocial interventions in pediatric oncology. Routine psychosocial screening of YACCS for HRQOL, anxiety, depression, and fatigue is recommended. Psychosocial interventions should be offered to YACCS proactively and focus primarily on perceived impact of cancer.

## Data Availability

The datasets generated during and/or analyzed during the current study are available from the corresponding author on reasonable request.
